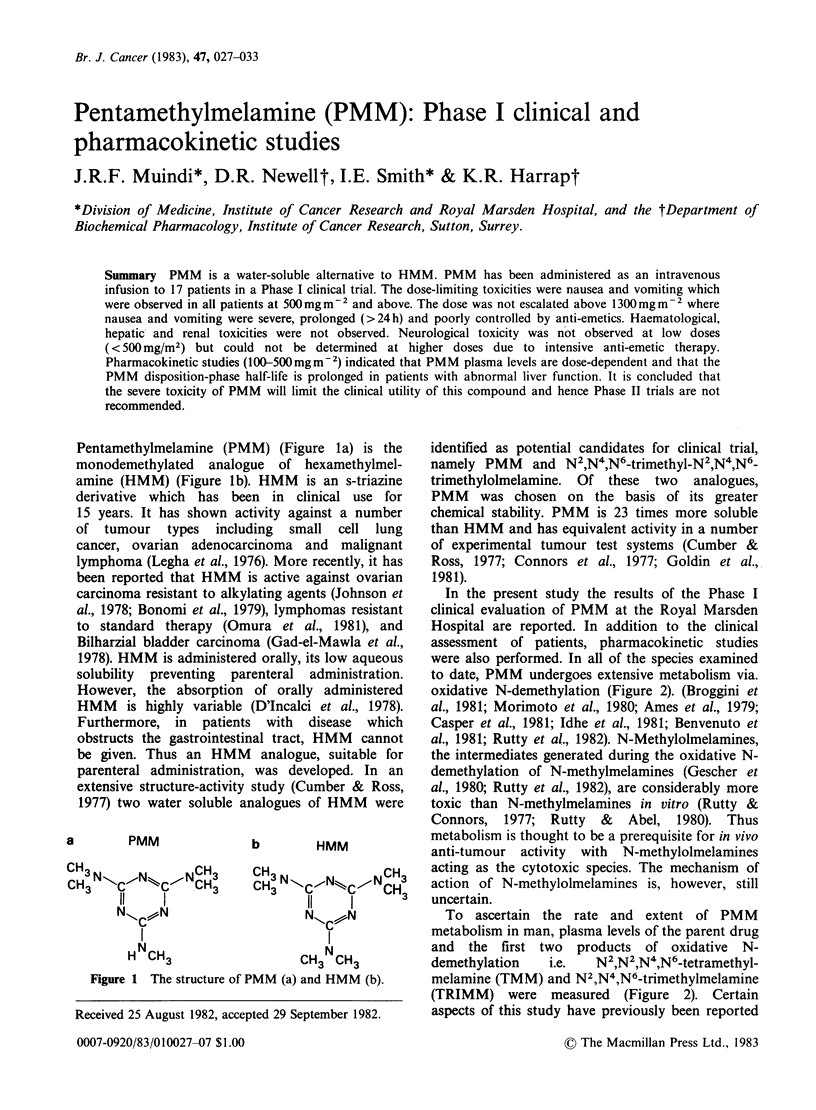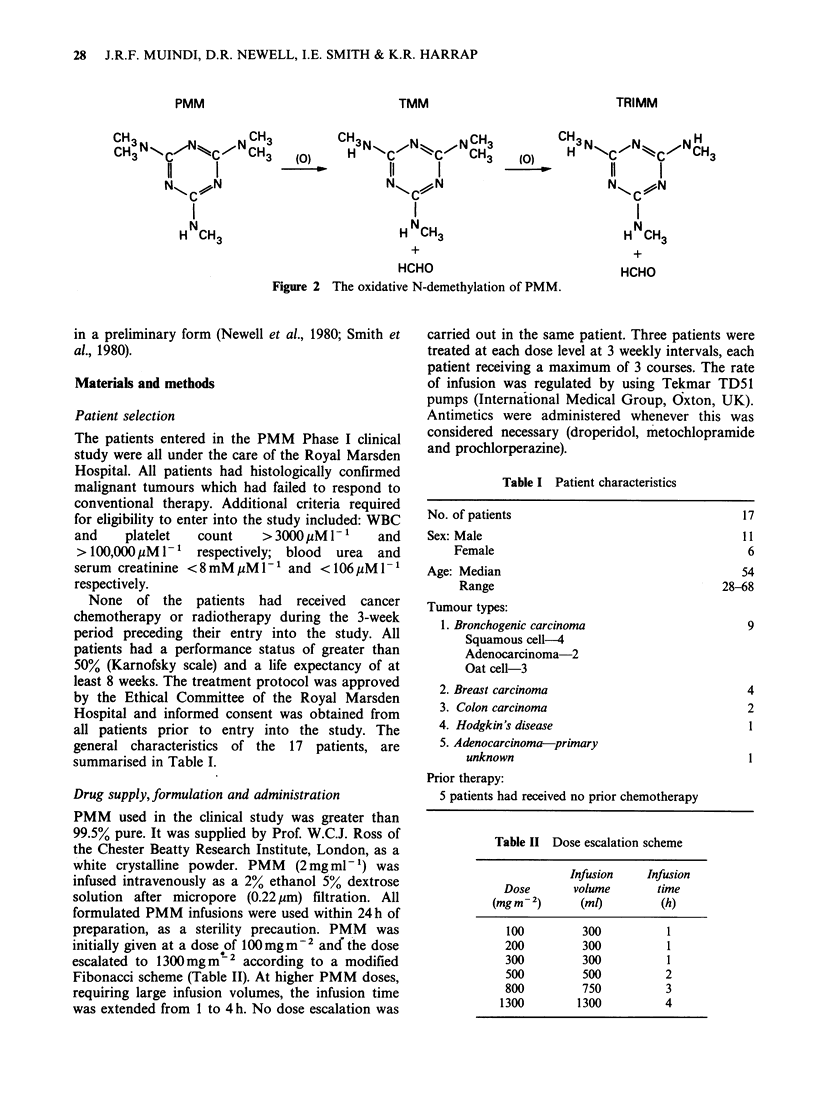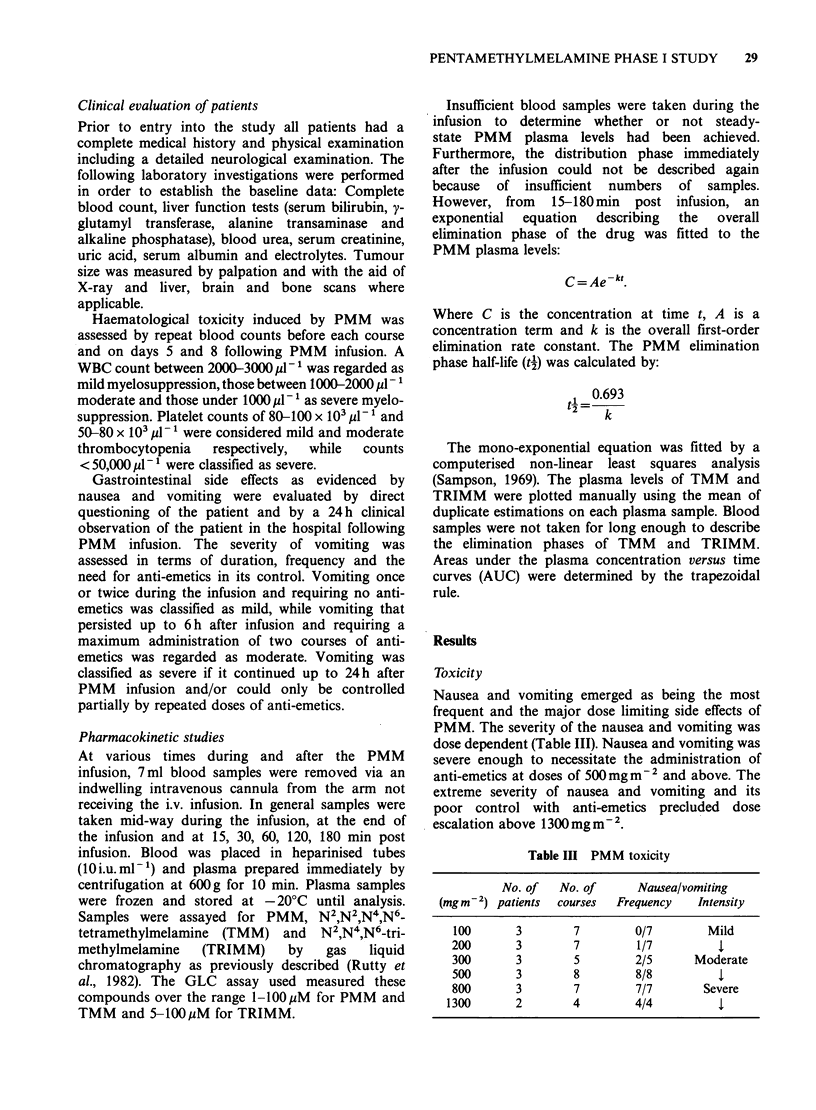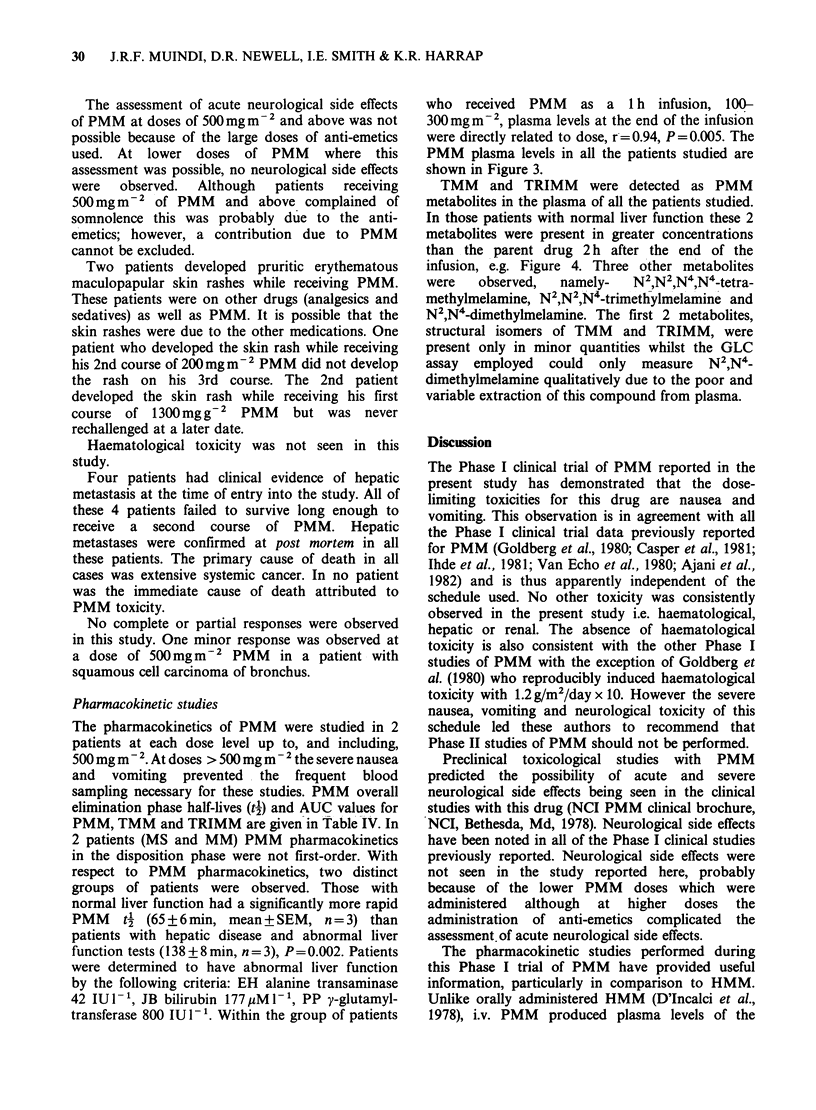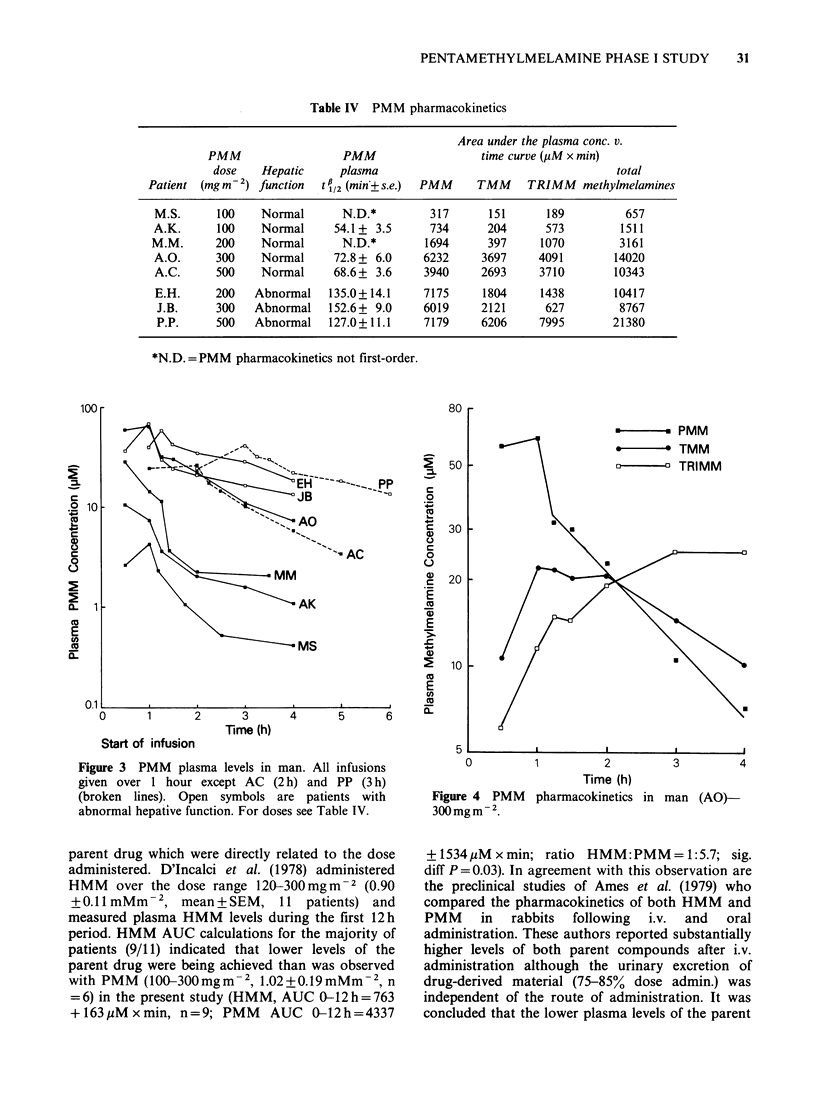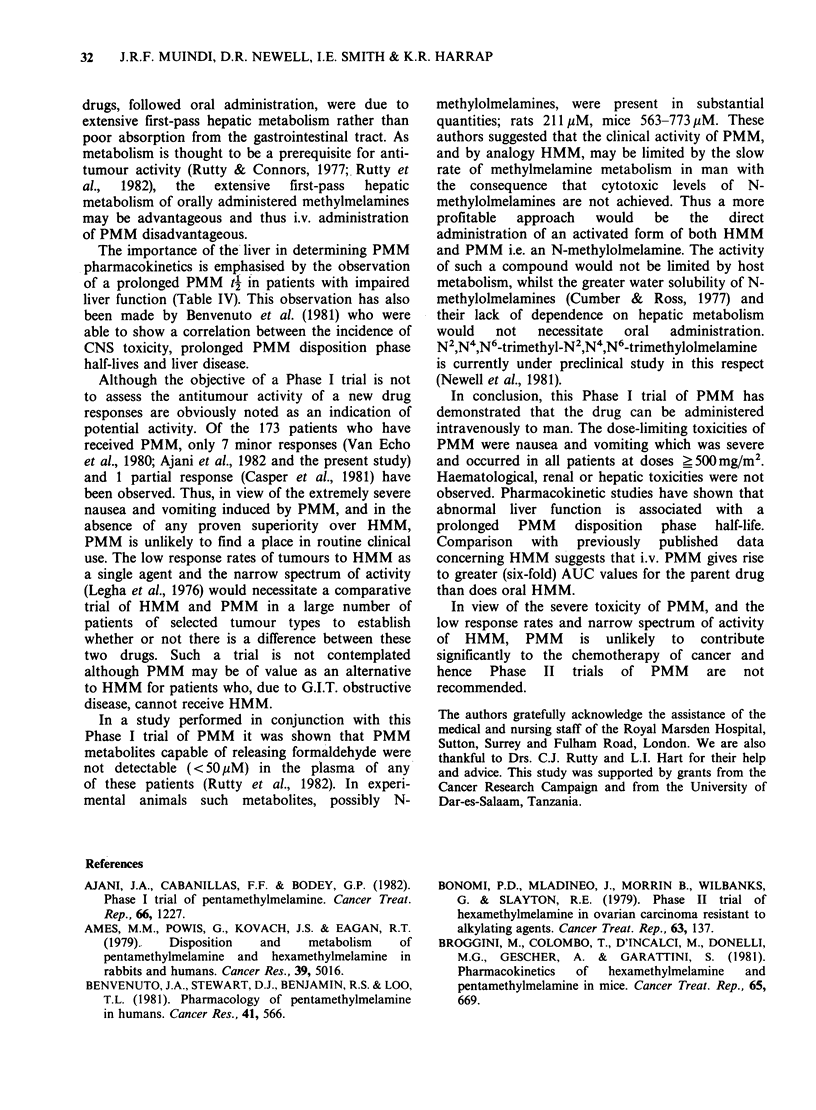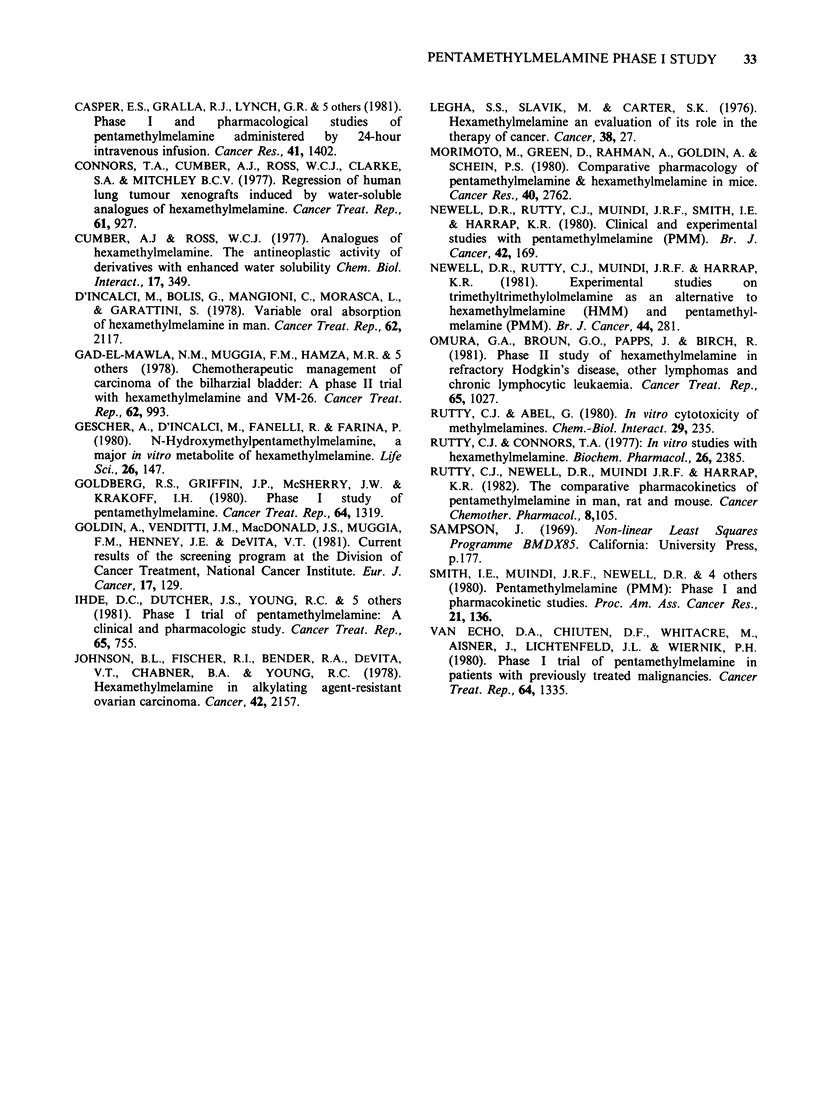# Pentamethylmelamine (PMM): Phase I clinical and pharmacokinetic studies.

**DOI:** 10.1038/bjc.1983.3

**Published:** 1983-01

**Authors:** J. R. Muindi, D. R. Newell, I. E. Smith, K. R. Harrap

## Abstract

PMM is a water-soluble alternative to HMM. PMM has been administered as an intravenous infusion to 17 patients in a Phase I clinical trial. The dose-limiting toxicities were nausea and vomiting which were observed in all patients at 500 mg m-2 and above. The dose was not escalated above 1300 mg m-2 where nausea and vomiting were severe, prolonged (greater than 24 h) and poorly controlled by anti-emetics. Haematological, hepatic and renal toxicities were not observed. Neurological toxicity was not observed at low doses (less than 500 mg/m2) but could not be determined at higher doses due to intensive anti-emetic therapy. Pharmacokinetic studies (100-500 mg m-2) indicated that PMM plasma levels are dose-dependent and that the PMM disposition-phase half-life is prolonged in patients with abnormal liver function. It is concluded that the severe toxicity of PMM will limit the clinical utility of this compound and hence Phase II trials are not recommended.


*Br. J. Cancer* (1983), **47**, 027–033

### Pentamethylmelamine (PMM): Phase I clinical and pharmacokinetic studies

J.R.F. Muindi*, D.R. Newell†, I.E. Smith* & K.R. Harrap†

**Division of Medicine, Institute of Cancer Research and Royal Marsden Hospital, and the †Department of Biochemical Pharmacology, Institute of Cancer Research, Sutton, Surrey.*

**Summary** PMM is a water-soluble alternative to HMM. PMM has been administered as an intravenous infusion to 17 patients in a Phase I clinical trial. The dose-limiting toxicities were nausea and vomiting which were observed in all patients at $500\,mg\,m^{-2}$ and above. The dose was not escalated above $1300\,mg\,m^{-2}$ where nausea and vomiting were severe, prolonged (>24 h) and poorly controlled by anti-emetics. Haematological, hepatic and renal toxicities were not observed. Neurological toxicity was not observed at low doses ($<500\,mg/m^2$) but could not be determined at higher doses due to intensive anti-emetic therapy. Pharmacokinetic studies ($100$–$500\,mg\,m^{-2}$) indicated that PMM plasma levels are dose-dependent and that the PMM disposition-phase half-life is prolonged in patients with abnormal liver function. It is concluded that the severe toxicity of PMM will limit the clinical utility of this compound and hence Phase II trials are not recommended.

Pentamethylmelamine (PMM) (Figure 1a) is the monodemethylated analogue of hexamethylmelamine (HMM) (Figure 1b). HMM is an s-triazine derivative which has been in clinical use for 15 years. It has shown activity against a number of tumour types including small cell lung cancer, ovarian adenocarcinoma and malignant lymphoma (Legha *et al.*, 1976). More recently, it has been reported that HMM is active against ovarian carcinoma resistant to alkylating agents (Johnson *et al.*, 1978; Bonomi *et al.*, 1979), lymphomas resistant to standard therapy (Omura *et al.*, 1981), and Bilharzial bladder carcinoma (Gad-el-Mawla *et al.*, 1978). HMM is administered orally, its low aqueous solubility preventing parenteral administration. However, the absorption of orally administered HMM is highly variable (D'Incalci *et al.*, 1978). Furthermore, in patients with disease which obstructs the gastrointestinal tract, HMM cannot be given. Thus an HMM analogue, suitable for parenteral administration, was developed. In an extensive structure-activity study (Cumber & Ross, 1977) two water soluble analogues of HMM were identified as potential candidates for clinical trial, namely PMM and $N^2,N^4,N^6$-trimethyl-$N^2,N^4,N^6$-trimethylolmelamine. Of these two analogues, PMM was chosen on the basis of its greater chemical stability. PMM is 23 times more soluble than HMM and has equivalent activity in a number of experimental tumour test systems (Cumber & Ross, 1977; Connors *et al.*, 1977; Goldin *et al.*, 1981).

**Figure 1** The structure of PMM (a) and HMM (b).

In the present study the results of the Phase I clinical evaluation of PMM at the Royal Marsden Hospital are reported. In addition to the clinical assessment of patients, pharmacokinetic studies were also performed. In all of the species examined to date, PMM undergoes extensive metabolism via. oxidative N-demethylation (Figure 2). (Broggini *et al.*, 1981; Morimoto *et al.*, 1980; Ames *et al.*, 1979; Casper *et al.*, 1981; Idhe *et al.*, 1981; Benvenuto *et al.*, 1981; Rutty *et al.*, 1982). N-Methylolmelamines, the intermediates generated during the oxidative N-demethylation of N-methylmelamines (Gescher *et al.*, 1980; Rutty *et al.*, 1982), are considerably more toxic than N-methylmelamines *in vitro* (Rutty & Connors, 1977; Rutty & Abel, 1980). Thus metabolism is thought to be a prerequisite for *in vivo* anti-tumour activity with N-methylolmelamines acting as the cytotoxic species. The mechanism of action of N-methylolmelamines is, however, still uncertain.

To ascertain the rate and extent of PMM metabolism in man, plasma levels of the parent drug and the first two products of oxidative N-demethylation i.e. $N^2,N^2,N^4,N^6$-tetramethylmelamine (TMM) and $N^2,N^4,N^6$-trimethylmelamine (TRIMM) were measured (Figure 2). Certain aspects of this study have previously been reported

Received 25 August 1982, accepted 29 September 1982.

0007-0920/83/010027-07 $1.00

© The Macmillan Press Ltd., 1983

**Figure 2** The oxidative N-demethylation of PMM.

in a preliminary form (Newell *et al.*, 1980; Smith *et al.*, 1980).

### Materials and methods

### *Patient selection*

The patients entered in the PMM Phase I clinical study were all under the care of the Royal Marsden Hospital. All patients had histologically confirmed malignant tumours which had failed to respond to conventional therapy. Additional criteria required for eligibility to enter into the study included: WBC and platelet count $>3000\,\mu M\,l^{-1}$ and $>100{,}000\,\mu M\,l^{-1}$ respectively; blood urea and serum creatinine $<8\,mM\,\mu M\,l^{-1}$ and $<106\,\mu M\,l^{-1}$ respectively.

None of the patients had received cancer chemotherapy or radiotherapy during the 3-week period preceding their entry into the study. All patients had a performance status of greater than 50% (Karnofsky scale) and a life expectancy of at least 8 weeks. The treatment protocol was approved by the Ethical Committee of the Royal Marsden Hospital and informed consent was obtained from all patients prior to entry into the study. The general characteristics of the 17 patients, are summarised in Table I.

### *Drug supply, formulation and administration*

PMM used in the clinical study was greater than 99.5% pure. It was supplied by Prof. W.C.J. Ross of the Chester Beatty Research Institute, London, as a white crystalline powder. PMM ($2\,mg\,ml^{-1}$) was infused intravenously as a 2% ethanol 5% dextrose solution after micropore ($0.22\,\mu m$) filtration. All formulated PMM infusions were used within 24 h of preparation, as a sterility precaution. PMM was initially given at a dose of $100\,mg\,m^{-2}$ and the dose escalated to $1300\,mg\,m^{-2}$ according to a modified Fibonacci scheme (Table II). At higher PMM doses, requiring large infusion volumes, the infusion time was extended from 1 to 4 h. No dose escalation was carried out in the same patient. Three patients were treated at each dose level at 3 weekly intervals, each patient receiving a maximum of 3 courses. The rate of infusion was regulated by using Tekmar TD51 pumps (International Medical Group, Oxton, UK). Antimetics were administered whenever this was considered necessary (droperidol, metochlopramide and prochlorperazine).

**Table I** Patient characteristics

| | |
|---|---|
| No. of patients | 17 |
| Sex: Male | 11 |
| Female | 6 |
| Age: Median | 54 |
| Range | 28–68 |
| Tumour types: | |
| 1. *Bronchogenic carcinoma* | 9 |
| Squamous cell—4 | |
| Adenocarcinoma—2 | |
| Oat cell—3 | |
| 2. *Breast carcinoma* | 4 |
| 3. *Colon carcinoma* | 2 |
| 4. *Hodgkin's disease* | 1 |
| 5. *Adenocarcinoma—primary unknown* | 1 |
| Prior therapy: | |
| 5 patients had received no prior chemotherapy | |

**Table II** Dose escalation scheme

| *Dose* ($mg\,m^{-2}$) | *Infusion volume* (*ml*) | *Infusion time* (*h*) |
|---|---|---|
| 100 | 300 | 1 |
| 200 | 300 | 1 |
| 300 | 300 | 1 |
| 500 | 500 | 2 |
| 800 | 750 | 3 |
| 1300 | 1300 | 4 |

*Clinical evaluation of patients*

Prior to entry into the study all patients had a complete medical history and physical examination including a detailed neurological examination. The following laboratory investigations were performed in order to establish the baseline data: Complete blood count, liver function tests (serum bilirubin, $\gamma$-glutamyl transferase, alanine transaminase and alkaline phosphatase), blood urea, serum creatinine, uric acid, serum albumin and electrolytes. Tumour size was measured by palpation and with the aid of X-ray and liver, brain and bone scans where applicable.

Haematological toxicity induced by PMM was assessed by repeat blood counts before each course and on days 5 and 8 following PMM infusion. A WBC count between 2000–3000 $\mu l^{-1}$ was regarded as mild myelosuppression, those between 1000–2000 $\mu l^{-1}$ moderate and those under 1000 $\mu l^{-1}$ as severe myelosuppression. Platelet counts of 80–100 $\times 10^3\ \mu l^{-1}$ and 50–80 $\times 10^3\ \mu l^{-1}$ were considered mild and moderate thrombocytopenia respectively, while counts $<50,000\ \mu l^{-1}$ were classified as severe.

Gastrointestinal side effects as evidenced by nausea and vomiting were evaluated by direct questioning of the patient and by a 24 h clinical observation of the patient in the hospital following PMM infusion. The severity of vomiting was assessed in terms of duration, frequency and the need for anti-emetics in its control. Vomiting once or twice during the infusion and requiring no anti-emetics was classified as mild, while vomiting that persisted up to 6 h after infusion and requiring a maximum administration of two courses of anti-emetics was regarded as moderate. Vomiting was classified as severe if it continued up to 24 h after PMM infusion and/or could only be controlled partially by repeated doses of anti-emetics.

*Pharmacokinetic studies*

At various times during and after the PMM infusion, 7 ml blood samples were removed via an indwelling intravenous cannula from the arm not receiving the i.v. infusion. In general samples were taken mid-way during the infusion, at the end of the infusion and at 15, 30, 60, 120, 180 min post infusion. Blood was placed in heparinised tubes (10 i.u. ml$^{-1}$) and plasma prepared immediately by centrifugation at 600 g for 10 min. Plasma samples were frozen and stored at −20°C until analysis. Samples were assayed for PMM, $N^2,N^2,N^4,N^6$-tetramethylmelamine (TMM) and $N^2,N^4,N^6$-trimethylmelamine (TRIMM) by gas liquid chromatography as previously described (Rutty *et al.*, 1982). The GLC assay used measured these compounds over the range 1–100 $\mu$M for PMM and TMM and 5–100 $\mu$M for TRIMM.

Insufficient blood samples were taken during the infusion to determine whether or not steady-state PMM plasma levels had been achieved. Furthermore, the distribution phase immediately after the infusion could not be described again because of insufficient numbers of samples. However, from 15–180 min post infusion, an exponential equation describing the overall elimination phase of the drug was fitted to the PMM plasma levels:

$$C = Ae^{-kt}.$$

Where $C$ is the concentration at time $t$, $A$ is a concentration term and $k$ is the overall first-order elimination rate constant. The PMM elimination phase half-life ($t\frac{1}{2}$) was calculated by:

$$t\tfrac{1}{2} = \frac{0.693}{k}$$

The mono-exponential equation was fitted by a computerised non-linear least squares analysis (Sampson, 1969). The plasma levels of TMM and TRIMM were plotted manually using the mean of duplicate estimations on each plasma sample. Blood samples were not taken for long enough to describe the elimination phases of TMM and TRIMM. Areas under the plasma concentration *versus* time curves (AUC) were determined by the trapezoidal rule.

### Results

*Toxicity*

Nausea and vomiting emerged as being the most frequent and the major dose limiting side effects of PMM. The severity of the nausea and vomiting was dose dependent (Table III). Nausea and vomiting was severe enough to necessitate the administration of anti-emetics at doses of 500 mg m$^{-2}$ and above. The extreme severity of nausea and vomiting and its poor control with anti-emetics precluded dose escalation above 1300 mg m$^{-2}$.

**Table III** PMM toxicity

| | *No. of* | *No. of* | *Nausea/vomiting* | |
|---|---|---|---|---|
| (*mg m*$^{-2}$) | *patients* | *courses* | *Frequency* | *Intensity* |
| 100 | 3 | 7 | 0/7 | Mild |
| 200 | 3 | 7 | 1/7 | ↓ |
| 300 | 3 | 5 | 2/5 | Moderate |
| 500 | 3 | 8 | 8/8 | ↓ |
| 800 | 3 | 7 | 7/7 | Severe |
| 1300 | 2 | 4 | 4/4 | ↓ |

The assessment of acute neurological side effects of PMM at doses of 500 mg m$^{-2}$ and above was not possible because of the large doses of anti-emetics used. At lower doses of PMM where this assessment was possible, no neurological side effects were observed. Although patients receiving 500 mg m$^{-2}$ of PMM and above complained of somnolence this was probably due to the anti-emetics; however, a contribution due to PMM cannot be excluded.

Two patients developed pruritic erythematous maculopapular skin rashes while receiving PMM. These patients were on other drugs (analgesics and sedatives) as well as PMM. It is possible that the skin rashes were due to the other medications. One patient who developed the skin rash while receiving his 2nd course of 200 mg m$^{-2}$ PMM did not develop the rash on his 3rd course. The 2nd patient developed the skin rash while receiving his first course of 1300 mg g$^{-2}$ PMM but was never rechallenged at a later date.

Haematological toxicity was not seen in this study.

Four patients had clinical evidence of hepatic metastasis at the time of entry into the study. All of these 4 patients failed to survive long enough to receive a second course of PMM. Hepatic metastases were confirmed at *post mortem* in all these patients. The primary cause of death in all cases was extensive systemic cancer. In no patient was the immediate cause of death attributed to PMM toxicity.

No complete or partial responses were observed in this study. One minor response was observed at a dose of 500 mg m$^{-2}$ PMM in a patient with squamous cell carcinoma of bronchus.

*Pharmacokinetic studies*

The pharmacokinetics of PMM were studied in 2 patients at each dose level up to, and including, 500 mg m$^{-2}$. At doses $>$500 mg m$^{-2}$ the severe nausea and vomiting prevented the frequent blood sampling necessary for these studies. PMM overall elimination phase half-lives ($t\frac{1}{2}$) and AUC values for PMM, TMM and TRIMM are given in Table IV. In 2 patients (MS and MM) PMM pharmacokinetics in the disposition phase were not first-order. With respect to PMM pharmacokinetics, two distinct groups of patients were observed. Those with normal liver function had a significantly more rapid PMM $t\frac{1}{2}$ (65 $\pm$ 6 min, mean $\pm$ SEM, $n=3$) than patients with hepatic disease and abnormal liver function tests (138 $\pm$ 8 min, $n=3$), $P=0.002$. Patients were determined to have abnormal liver function by the following criteria: EH alanine transaminase 42 IU l$^{-1}$, JB bilirubin 177 $\mu$M l$^{-1}$, PP $\gamma$-glutamyl-transferase 800 IU l$^{-1}$. Within the group of patients who received PMM as a 1 h infusion, 100–300 mg m$^{-2}$, plasma levels at the end of the infusion were directly related to dose, r = 0.94, $P=0.005$. The PMM plasma levels in all the patients studied are shown in Figure 3.

TMM and TRIMM were detected as PMM metabolites in the plasma of all the patients studied. In those patients with normal liver function these 2 metabolites were present in greater concentrations than the parent drug 2 h after the end of the infusion, e.g. Figure 4. Three other metabolites were observed, namely- $N^2$,$N^2$,$N^4$,$N^4$-tetramethylmelamine, $N^2$,$N^2$,$N^4$-trimethylmelamine and $N^2$,$N^4$-dimethylmelamine. The first 2 metabolites, structural isomers of TMM and TRIMM, were present only in minor quantities whilst the GLC assay employed could only measure $N^2$,$N^4$-dimethylmelamine qualitatively due to the poor and variable extraction of this compound from plasma.

### Discussion

The Phase I clinical trial of PMM reported in the present study has demonstrated that the dose-limiting toxicities for this drug are nausea and vomiting. This observation is in agreement with all the Phase I clinical trial data previously reported for PMM (Goldberg *et al.*, 1980; Casper *et al.*, 1981; Ihde *et al.*, 1981; Van Echo *et al.*, 1980; Ajani *et al.*, 1982) and is thus apparently independent of the schedule used. No other toxicity was consistently observed in the present study i.e. haematological, hepatic or renal. The absence of haematological toxicity is also consistent with the other Phase I studies of PMM with the exception of Goldberg *et al.* (1980) who reproducibly induced haematological toxicity with 1.2 g/m$^2$/day $\times$ 10. However the severe nausea, vomiting and neurological toxicity of this schedule led these authors to recommend that Phase II studies of PMM should not be performed.

Preclinical toxicological studies with PMM predicted the possibility of acute and severe neurological side effects being seen in the clinical studies with this drug (NCI PMM clinical brochure, NCI, Bethesda, Md, 1978). Neurological side effects have been noted in all of the Phase I clinical studies previously reported. Neurological side effects were not seen in the study reported here, probably because of the lower PMM doses which were administered although at higher doses the administration of anti-emetics complicated the assessment of acute neurological side effects.

The pharmacokinetic studies performed during this Phase I trial of PMM have provided useful information, particularly in comparison to HMM. Unlike orally administered HMM (D'Incalci *et al.*, 1978), i.v. PMM produced plasma levels of the

**Table IV** PMM pharmacokinetics

| *Patient* | *PMM dose* $(mg\,m^{-2})$ | *Hepatic function* | *PMM plasma* $t^{\beta}_{1/2}$ *(min±s.e.)* | *Area under the plasma conc. v. time curve (μM × min)* *PMM* | *TMM* | *TRIMM* | *total methylmelamines* |
|---|---|---|---|---|---|---|---|
| M.S. | 100 | Normal | N.D.* | 317 | 151 | 189 | 657 |
| A.K. | 100 | Normal | 54.1± 3.5 | 734 | 204 | 573 | 1511 |
| M.M. | 200 | Normal | N.D.* | 1694 | 397 | 1070 | 3161 |
| A.O. | 300 | Normal | 72.8± 6.0 | 6232 | 3697 | 4091 | 14020 |
| A.C. | 500 | Normal | 68.6± 3.6 | 3940 | 2693 | 3710 | 10343 |
| E.H. | 200 | Abnormal | 135.0±14.1 | 7175 | 1804 | 1438 | 10417 |
| J.B. | 300 | Abnormal | 152.6± 9.0 | 6019 | 2121 | 627 | 8767 |
| P.P. | 500 | Abnormal | 127.0±11.1 | 7179 | 6206 | 7995 | 21380 |

*N.D. = PMM pharmacokinetics not first-order.

**Figure 3** PMM plasma levels in man. All infusions given over 1 hour except AC (2 h) and PP (3 h) (broken lines). Open symbols are patients with abnormal hepatic function. For doses see Table IV.

**Figure 4** PMM pharmacokinetics in man (AO)—300 mg m$^{-2}$.

parent drug which were directly related to the dose administered. D'Incalci *et al.* (1978) administered HMM over the dose range 120–300 mg m$^{-2}$ (0.90 ±0.11 mMm$^{-2}$, mean±SEM, 11 patients) and measured plasma HMM levels during the first 12 h period. HMM AUC calculations for the majority of patients (9/11) indicated that lower levels of the parent drug were being achieved than was observed with PMM (100–300 mg m$^{-2}$, 1.02±0.19 mMm$^{-2}$, n = 6) in the present study (HMM, AUC 0–12 h = 763 + 163 μM × min, n = 9; PMM AUC 0–12 h = 4337 ±1534 μM × min; ratio HMM:PMM = 1:5.7; sig. diff $P$ = 0.03). In agreement with this observation are the preclinical studies of Ames *et al.* (1979) who compared the pharmacokinetics of both HMM and PMM in rabbits following i.v. and oral administration. These authors reported substantially higher levels of both parent compounds after i.v. administration although the urinary excretion of drug-derived material (75–85% dose admin.) was independent of the route of administration. It was concluded that the lower plasma levels of the parent drugs, followed oral administration, were due to extensive first-pass hepatic metabolism rather than poor absorption from the gastrointestinal tract. As metabolism is thought to be a prerequisite for antitumour activity (Rutty & Connors, 1977; Rutty *et al.*, 1982), the extensive first-pass hepatic metabolism of orally administered methylmelamines may be advantageous and thus i.v. administration of PMM disadvantageous.

The importance of the liver in determining PMM pharmacokinetics is emphasised by the observation of a prolonged PMM $t\frac{1}{2}$ in patients with impaired liver function (Table IV). This observation has also been made by Benvenuto *et al.* (1981) who were able to show a correlation between the incidence of CNS toxicity, prolonged PMM disposition phase half-lives and liver disease.

Although the objective of a Phase I trial is not to assess the antitumour activity of a new drug responses are obviously noted as an indication of potential activity. Of the 173 patients who have received PMM, only 7 minor responses (Van Echo *et al.*, 1980; Ajani *et al.*, 1982 and the present study) and 1 partial response (Casper *et al.*, 1981) have been observed. Thus, in view of the extremely severe nausea and vomiting induced by PMM, and in the absence of any proven superiority over HMM, PMM is unlikely to find a place in routine clinical use. The low response rates of tumours to HMM as a single agent and the narrow spectrum of activity (Legha *et al.*, 1976) would necessitate a comparative trial of HMM and PMM in a large number of patients of selected tumour types to establish whether or not there is a difference between these two drugs. Such a trial is not contemplated although PMM may be of value as an alternative to HMM for patients who, due to G.I.T. obstructive disease, cannot receive HMM.

In a study performed in conjunction with this Phase I trial of PMM it was shown that PMM metabolites capable of releasing formaldehyde were not detectable ($<50\,\mu$M) in the plasma of any of these patients (Rutty *et al.*, 1982). In experimental animals such metabolites, possibly N-methylolmelamines, were present in substantial quantities; rats $211\,\mu$M, mice $563–773\,\mu$M. These authors suggested that the clinical activity of PMM, and by analogy HMM, may be limited by the slow rate of methylmelamine metabolism in man with the consequence that cytotoxic levels of N-methylolmelamines are not achieved. Thus a more profitable approach would be the direct administration of an activated form of both HMM and PMM i.e. an N-methylolmelamine. The activity of such a compound would not be limited by host metabolism, whilst the greater water solubility of N-methylolmelamines (Cumber & Ross, 1977) and their lack of dependence on hepatic metabolism would not necessitate oral administration. $N^2$,$N^4$,$N^6$-trimethyl-$N^2$,$N^4$,$N^6$-trimethylolmelamine is currently under preclinical study in this respect (Newell *et al.*, 1981).

In conclusion, this Phase I trial of PMM has demonstrated that the drug can be administered intravenously to man. The dose-limiting toxicities of PMM were nausea and vomiting which was severe and occurred in all patients at doses $\geqq 500\,mg/m^2$. Haematological, renal or hepatic toxicities were not observed. Pharmacokinetic studies have shown that abnormal liver function is associated with a prolonged PMM disposition phase half-life. Comparison with previously published data concerning HMM suggests that i.v. PMM gives rise to greater (six-fold) AUC values for the parent drug than does oral HMM.

In view of the severe toxicity of PMM, and the low response rates and narrow spectrum of activity of HMM, PMM is unlikely to contribute significantly to the chemotherapy of cancer and hence Phase II trials of PMM are not recommended.

The authors gratefully acknowledge the assistance of the medical and nursing staff of the Royal Marsden Hospital, Sutton, Surrey and Fulham Road, London. We are also thankful to Drs. C.J. Rutty and L.I. Hart for their help and advice. This study was supported by grants from the Cancer Research Campaign and from the University of Dar-es-Salaam, Tanzania.

### References

AJANI, J.A., CABANILLAS, F.F. & BODEY, G.P. (1982). Phase I trial of pentamethylmelamine. *Cancer Treat. Rep.*, **66**, 1227.

AMES, M.M., POWIS, G., KOVACH, J.S. & EAGAN, R.T. (1979). Disposition and metabolism of pentamethylmelamine and hexamethylmelamine in rabbits and humans. *Cancer Res.*, **39**, 5016.

BENVENUTO, J.A., STEWART, D.J., BENJAMIN, R.S. & LOO, T.L. (1981). Pharmacology of pentamethylmelamine in humans. *Cancer Res.*, **41**, 566.

BONOMI, P.D., MLADINEO, J., MORRIN B., WILBANKS, G. & SLAYTON, R.E. (1979). Phase II trial of hexamethylmelamine in ovarian carcinoma resistant to alkylating agents. *Cancer Treat. Rep.*, **63**, 137.

BROGGINI, M., COLOMBO, T., D'INCALCI, M., DONELLI, M.G., GESCHER, A. & GARATTINI, S. (1981). Pharmacokinetics of hexamethylmelamine and pentamethylmelamine in mice. *Cancer Treat. Rep.*, **65**, 669.

CASPER, E.S., GRALLA, R.J., LYNCH, G.R. & 5 others (1981). Phase I and pharmacological studies of pentamethylmelamine administered by 24-hour intravenous infusion. *Cancer Res.*, **41**, 1402.

CONNORS, T.A., CUMBER, A.J., ROSS, W.C.J., CLARKE, S.A. & MITCHLEY B.C.V. (1977). Regression of human lung tumour xenografts induced by water-soluble analogues of hexamethylmelamine. *Cancer Treat. Rep.*, **61,** 927.

CUMBER, A.J & ROSS, W.C.J. (1977). Analogues of hexamethylmelamine. The antineoplastic activity of derivatives with enhanced water solubility *Chem. Biol. Interact.*, **17,** 349.

D'INCALCI, M., BOLIS, G., MANGIONI, C., MORASCA, L., & GARATTINI, S. (1978). Variable oral absorption of hexamethylmelamine in man. *Cancer Treat. Rep.*, **62,** 2117.

GAD-EL-MAWLA, N.M., MUGGIA, F.M., HAMZA, M.R. & 5 others (1978). Chemotherapeutic management of carcinoma of the bilharzial bladder: A phase II trial with hexamethylmelamine and VM-26. *Cancer Treat. Rep.*, **62,** 993.

GESCHER, A., D'INCALCI, M., FANELLI, R. & FARINA, P. (1980). N-Hydroxymethylpentamethylmelamine, a major *in vitro* metabolite of hexamethylmelamine. *Life Sci.*, **26,** 147.

GOLDBERG, R.S., GRIFFIN, J.P., McSHERRY, J.W. & KRAKOFF, I.H. (1980). Phase I study of pentamethylmelamine. *Cancer Treat. Rep.*, **64,** 1319.

GOLDIN, A., VENDITTI, J.M., MacDONALD, J.S., MUGGIA, F.M., HENNEY, J.E. & DeVITA, V.T. (1981). Current results of the screening program at the Division of Cancer Treatment, National Cancer Institute. *Eur. J. Cancer*, **17,** 129.

IHDE, D.C., DUTCHER, J.S., YOUNG, R.C. & 5 others (1981). Phase I trial of pentamethylmelamine: A clinical and pharmacologic study. *Cancer Treat. Rep.*, **65,** 755.

JOHNSON, B.L., FISCHER, R.I., BENDER, R.A., DEVITA, V.T., CHABNER, B.A. & YOUNG, R.C. (1978). Hexamethylmelamine in alkylating agent-resistant ovarian carcinoma. *Cancer*, **42,** 2157.

LEGHA, S.S., SLAVIK, M. & CARTER, S.K. (1976). Hexamethylmelamine an evaluation of its role in the therapy of cancer. *Cancer*, **38,** 27.

MORIMOTO, M., GREEN, D., RAHMAN, A., GOLDIN, A. & SCHEIN, P.S. (1980). Comparative pharmacology of pentamethylmelamine & hexamethylmelamine in mice. *Cancer Res.*, **40,** 2762.

NEWELL, D.R., RUTTY, C.J., MUINDI, J.R.F., SMITH, I.E. & HARRAP, K.R. (1980). Clinical and experimental studies with pentamethylmelamine (PMM). *Br. J. Cancer*, **42,** 169.

NEWELL, D.R., RUTTY, C.J., MUINDI, J.R.F. & HARRAP, K.R. (1981). Experimental studies on trimethyltrimethylolmelamine as an alternative to hexamethylmelamine (HMM) and pentamethylmelamine (PMM). *Br. J. Cancer*, **44,** 281.

OMURA, G.A., BROUN, G.O., PAPPS, J. & BIRCH, R. (1981). Phase II study of hexamethylmelamine in refractory Hodgkin's disease, other lymphomas and chronic lymphocytic leukaemia. *Cancer Treat. Rep.*, **65,** 1027.

RUTTY, C.J. & ABEL, G. (1980). *In vitro* cytotoxicity of methylmelamines. *Chem.-Biol. Interact.* **29,** 235.

RUTTY, C.J. & CONNORS, T.A. (1977): *In vitro* studies with hexamethylmelamine. *Biochem. Pharmacol.*, **26,** 2385.

RUTTY, C.J., NEWELL, D.R., MUINDI J.R.F. & HARRAP, K.R. (1982). The comparative pharmacokinetics of pentamethylmelamine in man, rat and mouse. *Cancer Chemother. Pharmacol.*, **8,**105.

SAMPSON, J. (1969). *Non-linear Least Squares Programme BMDX85*. California: University Press, p.177.

SMITH, I.E., MUINDI, J.R.F., NEWELL, D.R. & 4 others (1980). Pentamethylmelamine (PMM): Phase I and pharmacokinetic studies. *Proc. Am. Ass. Cancer Res.*, **21, 136.**

VAN ECHO, D.A., CHIUTEN, D.F., WHITACRE, M., AISNER, J., LICHTENFELD, J.L. & WIERNIK, P.H. (1980). Phase I trial of pentamethylmelamine in patients with previously treated malignancies. *Cancer Treat. Rep.*, **64,** 1335.